# Population genomics of pneumococcal carriage in Massachusetts children following introduction of PCV-13

**DOI:** 10.1099/mgen.0.000252

**Published:** 2019-02-19

**Authors:** Patrick K. Mitchell, Taj Azarian, Nicholas J. Croucher, Alanna Callendrello, Claudette M. Thompson, Stephen I. Pelton, Marc Lipsitch, William P. Hanage

**Affiliations:** ^1^​ Center for Communicable Disease Dynamics, Department of Epidemiology, T.H. Chan School of Public Health, Harvard University, Boston, MA, USA; ^2^​ MRC Centre for Global Infectious Disease Analysis, Department of Infectious Disease Epidemiology, Imperial College London, London W2 1PG, UK; ^3^​ Division of Pediatric Infectious Diseases, Maxwell Finland Laboratory for Infectious Diseases, Boston Medical Center, Boston, MA, USA

**Keywords:** *Streptococcus pneumoniae*, phylogenetics, PCV-13, population genomics, population dynamics, conjugate vaccines

## Abstract

The 13-valent pneumococcal conjugate vaccine (PCV-13) was introduced in the United States in 2010. Using a large paediatric carriage sample collected from shortly after the introduction of PCV-7 to several years after the introduction of PCV-13, we investigate alterations in the composition of the pneumococcal population following the introduction of PCV-13, evaluating the extent to which the post-vaccination non-vaccine type (NVT) population mirrors that from prior to vaccine introduction and the effect of PCV-13 on vaccine type lineages. Draft genome assemblies from 736 newly sequenced and 616 previously published pneumococcal carriage isolates from children in Massachusetts between 2001 and 2014 were analysed. Isolates were classified into one of 22 sequence clusters (SCs) on the basis of their core genome sequence. We calculated the SC diversity for each sampling period as the probability that any two randomly drawn isolates from that period belong to different SCs. The sampling period immediately after the introduction of PCV-13 (2011) was found to have higher diversity than preceding (2007) or subsequent (2014) sampling periods {Simpson’s *D* 2007: 0.915 [95 % confidence interval (CI) 0.901, 0.929]; 2011:  0.935 [0.927, 0.942]; 2014 :  0.912 [0.901, 0.923]}. Amongst NVT isolates, we found the distribution of SCs in 2011 to be significantly different from that in 2007 or 2014 (Fisher’s exact test *P*=0.018, 0.0078), but did not find a difference comparing 2007 to 2014 (Fisher’s exact test *P*=0.24), indicating greater similarity between samples separated by a longer time period than between samples from closer time periods. We also found changes in the accessory gene content of the NVT population between 2007 and 2011 to have been reduced by 2014. Amongst the new serotypes targeted by PCV-13, four were present in our sample. The proportion of our sample composed of PCV-13-only vaccine serotypes 19A, 6C and 7F decreased between 2007 and 2014, but no such reduction was seen for serotype 3. We did, however, observe differences in the genetic composition of the pre- and post-PCV-13 serotype 3 population. Our isolates were collected during discrete sampling periods from a small geographical area, which may limit the generalizability of our findings. Pneumococcal diversity increased immediately following the introduction of PCV-13, but subsequently returned to pre-vaccination levels. This is reflected in the distribution of NVT lineages, and, to a lesser extent, their accessory gene frequencies. As such, there may be a period during which the population is particularly disrupted by vaccination before returning to a more stable distribution. The persistence and shifting genetic composition of serotype 3 is a concern and warrants further investigation.

## Data Summary

Accession numbers and associated metadata for all isolates described in this study are provided in Table S1 (available in the online version of this article).

**Impact Statement**

Using genomic data from carriage pneumococcal isolates collected over a 14-year period spanning from shortly after the introduction of PCV-7 to four years after the introduction of PCV-13, we provide a detailed view of the effects of several years of conjugate vaccination on paediatric pneumococcal carriage in Massachusetts. We find that pneumococcal diversity spiked immediately following PCV-13 introduction before returning to pre-vaccination levels. This trend was reflected in both the clonal composition of the non-vaccine type population and the gene content of the population. Together, these findings indicate that the period immediately following PCV-13 introduction saw changes to the population of non-vaccine type pneumococci, but that over time the population trended back towards its pre-vaccination state. Additionally, we examined how the population of serotype 3 pneumococci changed following PCV-13 introduction, finding no evidence for a decrease in serotype 3 carriage, in contrast to the other new vaccine serotypes. However, we do find a shift in the genetic makeup of the serotype 3 population following PCV-13 introduction. This work provides insight into how the pneumococcal population responds to the intense selective pressure exerted by conjugate vaccine and shows evidence of the population stabilizing over time.

## Introduction

*Streptococcus pneumoniae* is a common bacterial colonizer of the human nasopharynx, particularly among children [1]. In Massachusetts, it has consistently been found in approximately 30 % of children under the age of 7 years between 2001 and 20 11 [2]. While colonization rarely progresses beyond asymptomatic carriage, the ubiquity of the pneumococcus leads to a substantial burden of disease, causing an estimated 4 million disease episodes, including 445 000 hospitalizations and 22 000 deaths in the United States in 2004 [3].

Conjugate vaccination has been a major advance in reducing pneumococcal disease. The seven-valent pneumococcal conjugate vaccine (PCV-7), introduced in the United States in 2000, was highly effective in reducing overall rates of pneumococcal disease, as vaccine type (VT) pneumococci were responsible for the vast majority of cases [4–6]. Carriage of vaccine serotypes also declined, although overall carriage prevalence remained roughly constant due to serotype replacement [2, 7, 8].

Despite lower overall rates of pneumococcal disease, increases were seen in the incidence of disease due to the replacement non-vaccine type (NVT) population. Serotype 19A in particular became a significant cause of invasive disease [5, 9, 10]. The 13-valent vaccine (PCV-13), introduced in 2010, extended coverage to six additional serotypes, including 19A, beyond those included in PCV-7, and has resulted in further reductions in pneumococcal disease [11]. As with PCV-7, however, overall carriage prevalence has not changed substantially [2]. Worryingly, serotype 3, a highly invasive serotype included in PCV-13, appears not to have declined as compared with the other newly added serotypes [2, 11–14]. Given the potential for disease to arise both from replacement NVTs and from persistent VTs, it remains important to monitor changes to the pneumococcal carriage population.

Paediatric pneumococcal carriage in Massachusetts has been extensively studied since shortly after the introduction of PCV-7 [7]. The effects of vaccination can be seen both in the prevalence of specific lineages as well as in broader population metrics. The apparent effects of vaccination are variable depending on how the population is characterized and the timescale over which it is examined. Serotype diversity was found to have increased then stabilized following the introduction of PCV-7 [15], reflecting the selective impact of vaccines and the period while carriage replacement was taking place. Interestingly, minimal changes were found when comparing the presence and absence of specific pneumococcal genes in this population between 2001 and 2007, suggesting that the overall genetic composition of the population was not much changed other than in one of the loci conferring vaccine serotype 6B [16]. Another study considering multilocus sequence type (MLST) profiles found no significant change in diversity or population composition in the immediate aftermath of PCV-13 introduction [17]. With more time elapsed since PCV-13 introduction, it is possible to evaluate the longer-term effects of this vaccine.

Here we examine population-scale genetic changes in carriage pneumococci amongst children in Massachusetts since the introduction of PCV-13. Using genomic sequencing data for isolates collected between 2000 and 2014, we analyse alterations to the clonal composition, defined on the basis of core genome variability, and gene content of the pneumococcal NVT population following the introduction of PCV-13. Additionally, we evaluate whether serotype 3 pneumococci have declined and how they have changed through this time period.

## Methods

### Sample collection

Pneumococcal isolates were collected from nasopharyngeal swabs of children under the age of 7 years old being seen at a participating primary care provider in communities throughout Massachusetts, USA. Samples were collected between October and April of 2000–01, 2003–04, 2006–07, 2008–09, 2010–11 and 2013–14. Each sampling season is referred to by the later year. The number of participating provider sites varied between eight and 16 per sampling season as previously described [2, 7, 8]. Pneumococcal genomes from the 2001, 2004 and 2007 sampling periods were previously published and read data for these were obtained from ENA [16]. Isolates from 2009 to 2014 were sequenced from NexteraXT genomic libraries analysed on an Illumina MiSeq to produce paired-end 2×150 bp reads with a minimum depth of coverage of 30×. Sequencing data generated for this study have been deposited in the NCBI Sequence Read Archive under BioProject PRJNA437292. Accession numbers and isolate metadata are listed in Table S1.

### Genomic processing

Draft assemblies were constructed using SPAdes v3.10 and annotated using Prokka v1.11 [18, 19]. Assemblies not between 1.9 and 2.3 Mb were excluded from further analysis, as were those that produced fewer than 1900 annotated coding sequences (CDS). Roary v3.10.0 was then used to identify core (present in >99 % of isolates) and accessory genes and to generate a core gene alignment [20].

### Typing

Serotype was identified using the Quellung reaction as previously described and reported for all but the 2014 sample [16, 17, 21]. Serotypes were checked using SRST2 v0.2.0 and a database was constructed from 91 published sequences of the pneumococcal capsule biosynthetic locus [22–24].

### Phylogenetic analysis

The core genome alignment generated by Roary was used to construct a phylogeny using FastTree v2.1.10 [25]. In order to identify clusters of related sequences [sequence clusters (SCs)], three iterations of hierBAPS were run on the core genome alignment, setting the maximum cluster depth to 1 and maximum number of clusters to 30, 40 and 50 [26].

### Sequence cluster diversity

In order to determine the potential effect of PCV-13 on diversity in this population, we calculated Simpson’s *D* for each sampling period, for SCs. This value, which represents the probability that two randomly drawn isolates from a given sampling period belong to different SCs, was calculated as $D=\frac{N}{N-1}(1-\sum_{i=1}^{m}x_{i}^{2})$, where $x=\frac{n_{i}}{N}$, the fraction of isolates in that year belonging to SC i and $\frac{N}{N-1}$ is a correction for finite sample size [27]. Following an earlier analysis of serotype diversity in this population, Welch’s *t*-test was used to compare the 2007 and 2011 populations and the 2011 and 2014 populations in order to test whether SC diversity changed following the introduction of PCV-13 [15]. The polyphyletic SC was excluded from these calculations.

An increase in diversity would be expected if common lineages become more rare and rare lineages become more common. To estimate the expected change in diversity we would observe if there were a smooth transition between the 2007 and 2014 population, a series of composite diversities were calculated in which the proportion belonging to each SC was a weighted combination of the 2007 and 2014 value for that SC, with the weights for the two years summing to 1. The sample size correction factor, $\frac{N}{N-1}$, was similarly weighted.

The proportion of the population belonging to each SC and their rank order in the population were determined. As diversity increases, the shape of this distribution would be expected to flatten, with the most common lineages decreasing and the least common lineages increasing [28]. In order to compare this distribution from the 2007 and 2014 sampling periods with that from 2011, the frequency of each SC was plotted against its rank and overlaid with the distribution from 2011. In order to determine which SCs became more or less common following the introduction of PCV-13, we conducted a Fisher’s exact test for each SC comparing its frequency between the 2007 and 2014 samples.

### NVT composition

To determine the clonal composition of the pre- and post-PCV-13 NVT population, the proportion of the NVT population belonging to each of the SCs identified by hierBAPS was calculated for 2007, 2011 and 2014. For the purpose of these analyses, serotype 6C was considered a PCV-13 type due to its cross-reactivity with serotype 6A [29, 30]. Fisher’s exact test was used to determine whether these proportions varied between each pairwise combination of these three sampling periods.

We then sought to determine if the gene content of the NVT population varied between sampling periods before and after the introduction of PCV-13. Logistic models were used to evaluate the extent to which individual genes became more or less common between 2007 and 2014, as well as between 2007 and 2011. Genes were excluded if they were universally present or absent in either sampling period or present or absent in fewer than five isolates between the three sampling periods. For the set of genes included in both models, we calculated a linear fit comparing the regression coefficients corresponding to the time periods from 2007 to 2011 and 2007 to 2014.

In order to determine whether changes in the gene content of the NVT population from 2007 to 2011 continued, stabilized or reversed from 2011 to 2014, we compared the observed data to hypothetical scenarios in which the 2014 population was purely reflective of the population from either of the earlier sampling periods. To do this, we drew with replacement a sample of the same size as the 2014 population from either the 2007 or the 2011 population. Twenty resampled populations were generated from each of 2007 and 2011, then used in place of the true 2014 population in the previous regression analyses. This process was repeated for an additional 20 resampled populations drawn from the true 2014 population in order to gauge its variability. This enabled us to evaluate the gene content of the 2014 population in relation to what would be expected if there was no overall change either from 2007 or from 2011.

### Evaluation of serotype 3

Previous studies have noted that PCV-13 may not be as effective against serotype 3 as it is against the other serotypes included [2, 11, 13, 14]. We compared the proportion of the pneumococcal population composed of serotype 3 between 2007, 2011 and 2014 in relation to the other three PCV-13 serotypes present in our sample, 19A, 7F and 6C. We identified the MLST profile of serotype 3 isolates as previously described [16]. We then used RAxML to construct a phylogenetic tree based on the core genome of serotype 3 isolates to determine if the pre- and post-PCV-13 populations were genetically distinct [31]. To assess nucleotide and amino acid variation among capsular polysaccharide (CPS) loci, we mapped reads to the *S. pneumoniae* OXC141 serotype 3 reference strain (NC_017592) using SMALT v0.7.6. SNPs were identified using SAMtools v1.3.1 [32]. The CPS region spanning nucleotides 343 104–356 408 (*dexB – aliA*) was abstracted and investigated for mutations. Further, RAxML was used to construct a phylogeny of the CPS region.

## Results

### Sample

**Fig. 1.**
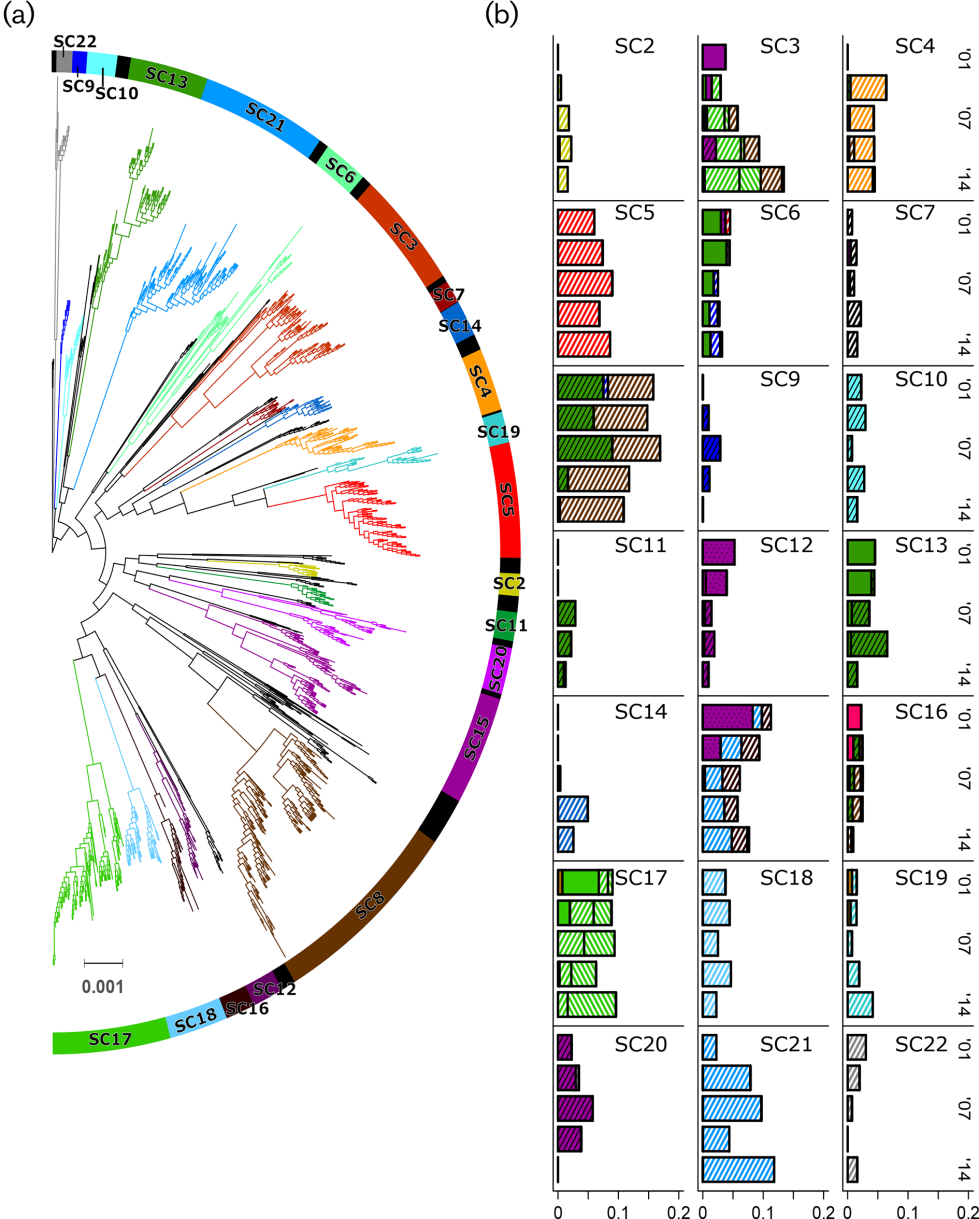
(a) Core genome phylogeny with SCs denoted by colour. (b) Proportion of population in each sampling period composed of each SC, with shading indicating serotype. Solid colours are PCV-7 type, solid colours with black hatching are PCV-13, and white with coloured hatching are not covered by either. Serotype 6A is dotted as it is cross-reactive with 6B, a PCV-7 type, but is itself included in PCV-13.

A total of 1352 isolates were included in the final analysis. The core genome consists of 1000 genes found in at least 99 % of isolates, producing an alignment 885 kb in length. A total of 10 941 genes were identified. Setting the maximum number of hierBAPS clusters to 30 and 40 produced identical results, with 21 clusters identified. With the maximum number of clusters set to 50, an additional cluster was identified and another cluster was expanded. This resulted in 22 SCs, 21 of which were monophyletic and ranged in size from 14 to 177 isolates. The other, SC1, contained 150 isolates belonging to multiple small clades or individual leaves throughout the tree and should be interpreted as containing all lineages that could not be grouped, other than on the basis of their lack of similarity to any other cluster (Fig. 1).

### Diversity

**Fig. 2.**
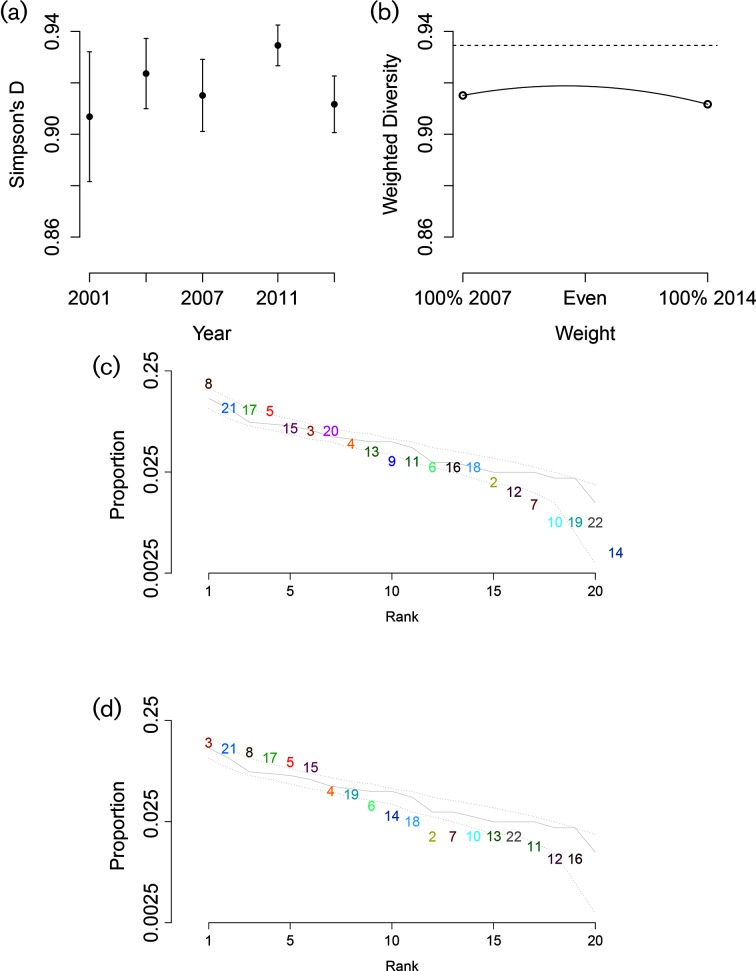
(a) Simpson’s diversity of SCs, excluding the polyphyletic cluster, for each sampling period. (b) Diversity calculated for hypothetical composites of 2007 and 2014 populations, with 2011 diversity shown as a dashed line. (c, d) Proportion of population in (c) 2007 and (d) 2014 composed of each SC, ordered by frequency. The grey lines (c, d) correspond to the distribution from 2011, with dotted grey lines representing 95 % of values from 10 000 random samples drawn from the 2011 population.

SC diversity was calculated for each year using Simpson’s *D*, excluding the polyphyletic cluster SC1. Diversity was significantly higher in 2011, the first sampling period following the introduction of PCV-13, than it was in either 2007 or 2014, the adjacent periods for which data were available (2007 *P*=0.018, 2014 *P*=0.00098) (Fig. 2a). A similar increase was observed after the introduction of PCV-7. The weighted diversity estimate displayed the expected increase over either the 2007 or the 2014 values, but was never as high as the diversity calculated for 2011 (Fig. 2b).

After Bonferroni correction, only three SCs (SC3, SC9 and SC20) changed significantly in their share of the pneumococcal population between 2007 and 2014 (Fisher’s exact test *P*=0.0021, 0.0022 and 4.5×10^−6^, respectively). SC3 became more common, increasing from 5.8 % of the 2007 sample to 13.4 % of the 2014 sample, with serogroups 23 and 15 coming to predominate over serogroup 6. Both SCs 9 and 20 are primarily composed of serotypes against which PCV-13 afforded protection (7F and 6C, respectively) and were completely absent from the 2014 sample.

The overall shape of the frequency distribution was slightly flatter in 2011 as compared to 2007 and 2014, as would be expected from the higher diversity in that sampling period. Relatively rare SCs in particular were more common in the 2011 sample than in the adjacent periods (Fig. 2c, d).

### NVT composition

**Fig. 3.**
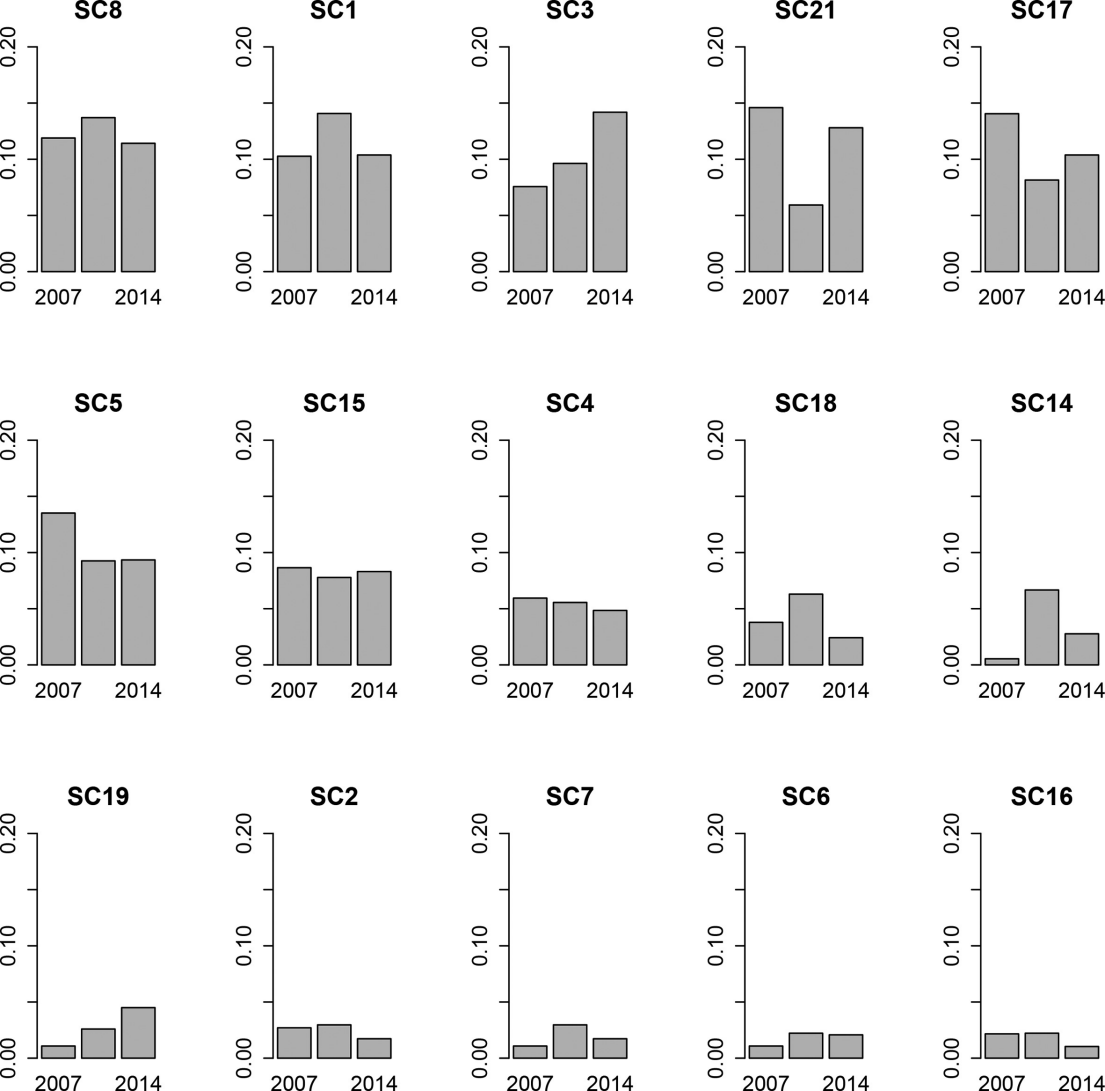
Proportion of the NVT population (i.e. those serotypes not included in PCV-13) comprising each SC. Two additional SCs, SC11 and SC20, had a single NVT isolate and were excluded from this plot.

Non-PCV-13 types increased from 66.5 % of the pneumococcal population in the 2007 sampling period to 92.3 % in the 2014 sampling period. Fifteen SCs had at least one NVT isolate. There was no significant difference between the SC distribution amongst NVTs in 2007 and 2014 (Fisher’s exact test *P*=0.24). There was, however, a significant difference between 2007 and 2011 (*P*=0.0018) and between 2011 and 2014 (*P*=0.0078), indicating a bounce-back effect in which the population was disrupted in 2011 but returned to its pre-vaccination state by 2014. Correspondingly, many of the common SCs that showed a distinct increase in their prevalence in the NVT population between 2007 and 2011 decreased from 2011 to 2014 while those that decreased between 2007 and 2011 increased from 2011 to 2014 (Fig. 3).

**Fig. 4.**
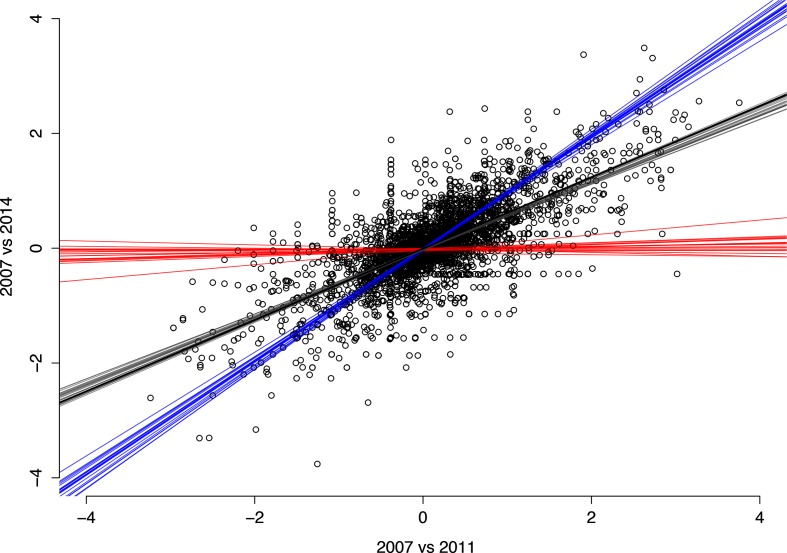
Regression coefficients comparing gene content of the NVT population from 2007 to 2011 and 2014. Black circles correspond to the coefficients with individual genes, with a linear fit to the data shown in black. Fits in which a hypothetical 2014 population was drawn from either the 2007, 2011 or 2014 population are shown in red, blue and grey, respectively.

This bounce-back is partially reflected by the trend in gene content over time. The linear fit comparing the 2007–2011 and 2007–2014 regression coefficients for each gene had a slope of 0.62, indicating less overall change between 2007 and 2014 than between 2007 and 2011. This slope fell between those from hypothetical 2014 populations drawn from either 2007 or 2011, which clustered around a slope of 0 and 1, respectively (Fig. 4). This indicates that while the direction in which genes changed in frequency from 2007 to 2011 was generally preserved through 2014, the trend was partially counteracted between 2011 and 2014 with genes returning closer to their 2007 levels prior to the introduction of PCV-13.

### Persistence of serotype 3

**Fig. 5.**
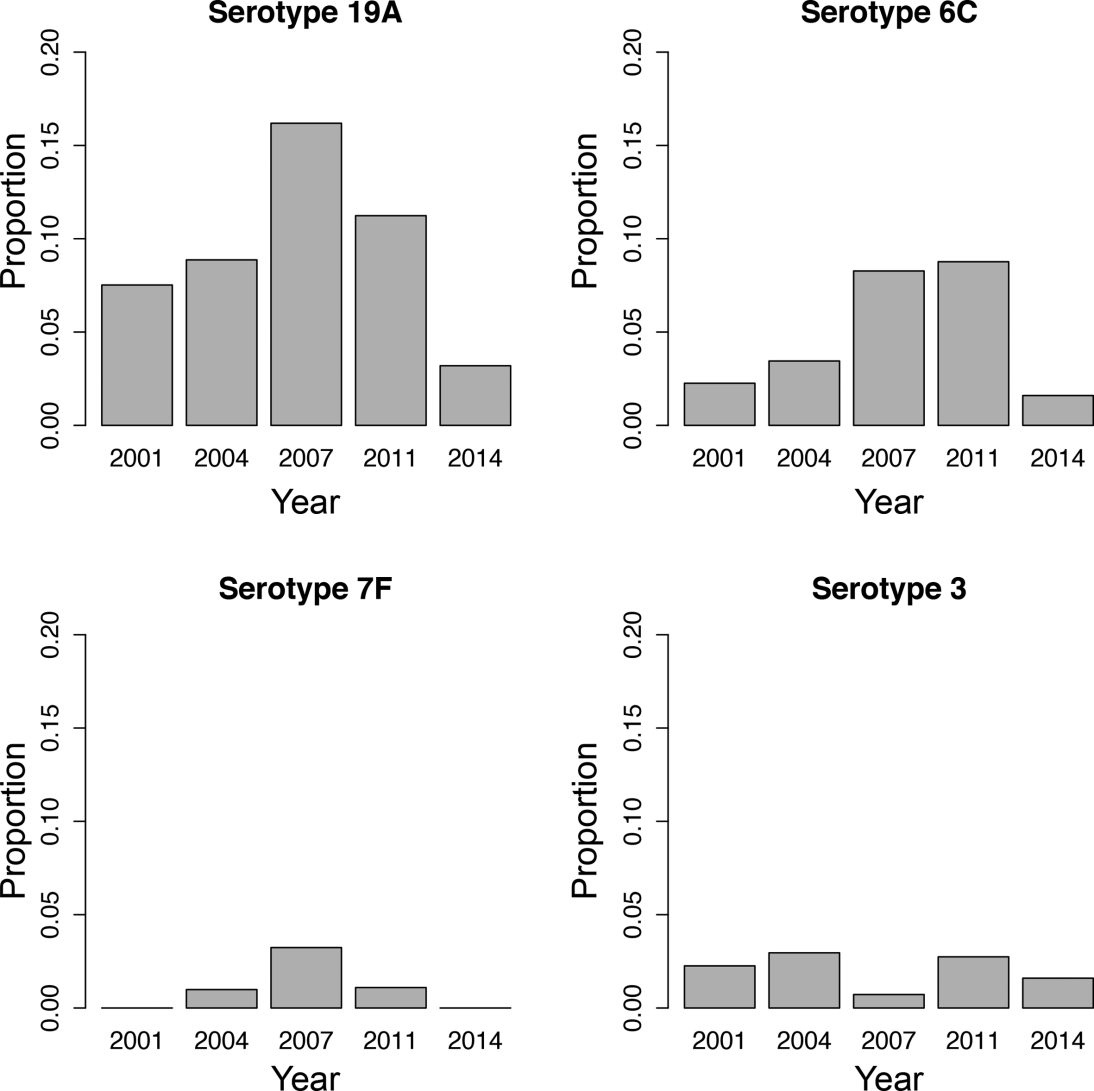
Proportion of the population in each sampling period comprising the serotypes included in PCV-13 but not PCV-7. As a note, PCV-7 was introduced in the United States in 2000 and PCV-13 was introduced in 2010.

In order to evaluate whether the new serotypes included in PCV-13 decreased following its introduction, we conducted a Fisher’s exact test comparing the 2007 and 2014 carriage share of serotypes 19A, 6C, 7F and 3. While serotypes 19A, 6C and 7F all showed significant reductions between the two time periods (*P*<0.0001, *P*=0.00014 and *P*=0.0011, respectively), serotype 3 had no such change (*P*=0.46) (Fig. 5).

**Fig. 6.**
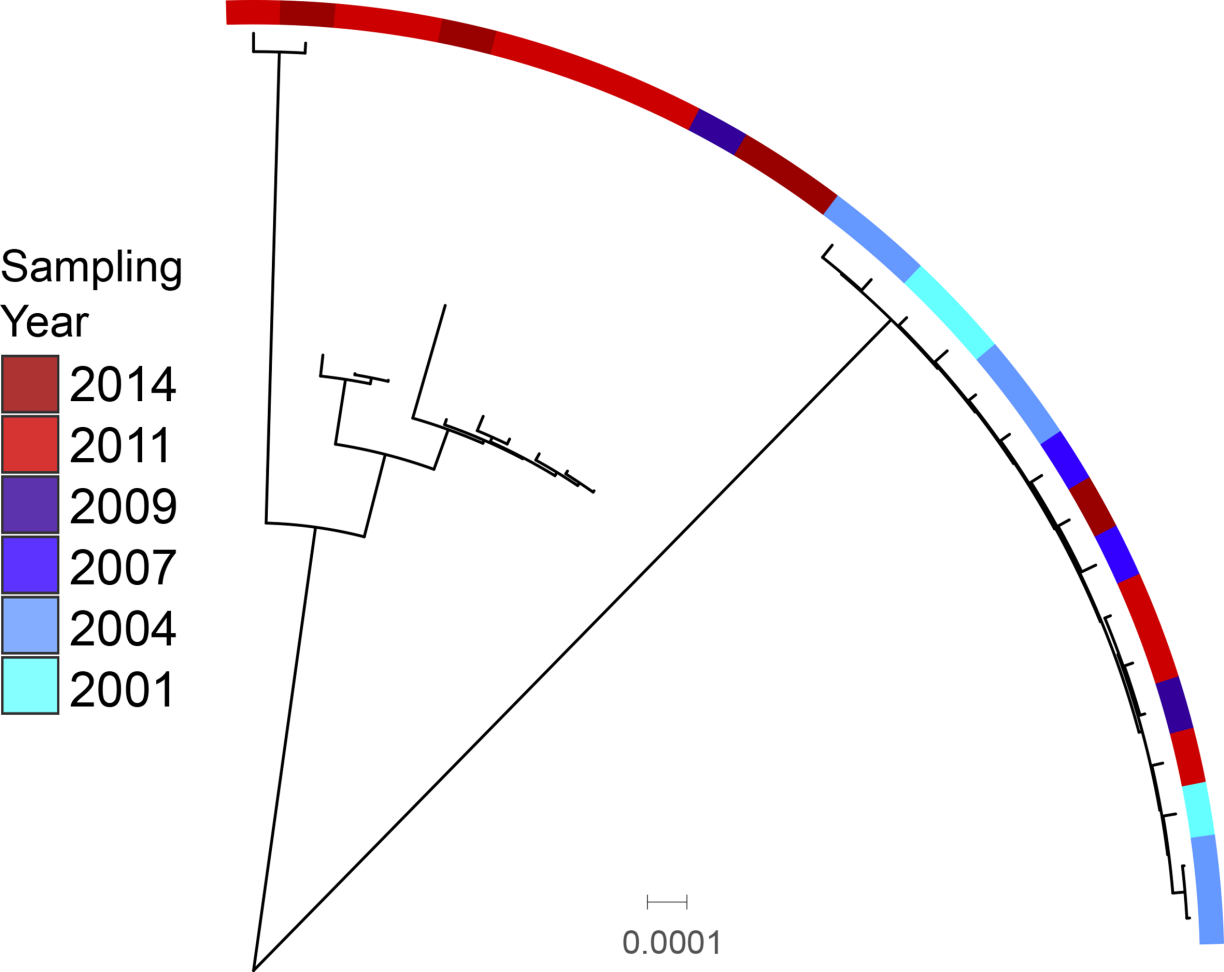
Serotype 3 maximum likelihood phylogeny, with sampling period shown by colour. Scale bar represents genetic distance. Isolates collected before the introduction of PCV-13, shown in blue, are found primarily on one monophyletic clade of the tree, while post-introduction isolates, in red, are primarily on the other.

To test if the persistence of serotype 3 may be related to some genetic factor, we assessed population structure and CPS nucleotide variation. All of the serotype 3 isolates clustered in the same SC and were MLST sequence type (ST) 180 belonging to the Netherlands 3–31 (PMEN31) clone CC180. While all isolates clustered into the same SC, there was a distinct bifurcation in the phylogeny (Fig. 6). Of the 28 serotype 3 isolates, 16 fell into one subclade and 12 into the other. In the larger subclade, four isolates (25 %) are from 2011 or 2014, after the introduction of PCV-13. The other subclade contains 11 (92 %) post-PCV-13 isolates (χ^2^ *P*=0.0018). Further assessment of CPS showed low nucleotide diversity [mean pairwise SNP distance: 1.5 (se 0.7)] and only four polymorphic amino acids, none of which segregated the subclades. However, the CPS phylogeny recapitulated the bifurcation in the core genome phylogeny, with all isolates belonging to the post-PCV-13 subclade displaying as highly clustered.

## Discussion

Here we have analysed a sample of carriage pneumococci collected in Massachusetts between the winters of 2000–01 and 2013–14, focusing primarily on changes occurring following the introduction of PCV-13. Using genomic data, we find that the NVT population in the most recent sampling period more closely reflects that of our last full pre-PCV-13 sample than our first post-PCV-13 sample. This suggests a return to equilibrium following disruption by vaccine, which is consistent with observations made following the introduction of PCV-7 in the same population [33], but now with the added resolution offered by genomic data. We also find that serotype 3 CC180 has been more persistent than other serotypes added for PCV-13, but a different subclade of this lineage now predominates.

Given the value of being able to predict the composition of the pneumococcal population following PCV use, the pattern observed amongst the NVTs is quite interesting. Our 2014 sample appears to be broadly a reflection of the 2007 sample, but 2011 is unlike either. As such, it is possible that the pre-vaccine NVT population may be a good predictor of the post-vaccine population, but that the disruption caused by vaccine introduction can temporarily interrupt this pattern. Some of this could be due to variation in the age of children who have been vaccinated, which should increase over time as vaccinated children age. The observed increase in SC diversity in the immediate post-vaccine period, with the most common lineages making up a smaller proportion of the total population, may provide an enhanced opportunity for rarer lineages to increase. Considering this scenario, lineages such as SCs 3, 14 and 19 (serotypes 23A/15BC, 21 and 33F, respectively) may have a similar trajectory to that of serotype 19A ST320 after PCV-7 [5, 9, 10, 34]. It has also recently been suggested that negative frequency-dependent selection on elements of the accessory genome could be responsible for structuring the pneumococcal population at both spatial and temporal scales [35]. Further observation will help to determine the role of this and whether these or other lineages become more substantial contributors to both carriage and invasive disease.

Previous studies have indicated that PCV-13 may not be as effective against serotype 3 as it is against other included serotypes [2, 11, 13, 14]. The shift we observed in the serotype 3 CC180 population following the introduction of PCV-13 may reflect a similar phenomenon to that leading to the recognition of serotype 6C as distinct from 6A following the introduction of PCV-7 [36, 37]. The dominant lineage pre-PCV-13 was also more homogeneous (i.e. less diverse) than the post-vaccination population, so it is possible that the immunity generated against serotype 3 by PCV-13 is narrowly tailored to that subset of the population, although a recent analysis of a global collection of over 300 serotype 3 isolates, including those described in this paper, did not find evidence to support a connection between PCV usage and serotype 3 clade composition [38]. Non-capsular antigenic variation and antibiotic resistance, however, were suggested as contributors to the post-PCV-13 success of this lineage. Specifically, a large proportion of the emergent clade harboured a *Tn916* transposon conferring resistance to tetracycline and macrolides.

The response of the pneumococcal population to serotype-targeting conjugate vaccines may also provide insights for other pathogens for which vaccines have been targeted at or differentially affect a subset of their population. The efficacy of the RTS,S malaria vaccine appears to be partially dependent on how well the circumsporozoite protein of a given *Plasmodium* type matches that in the vaccine [39]. There has also been interest in understanding how the strain dynamics and epidemiology of meningococcal disease caused by the bacteria *Neisseria meningitidis* will be affected by the rollout of vaccinations against a variety of serogroups [40, 41]. While each of these disease systems is different, there is some potential for findings in one to inform hypotheses for how others will behave.

Pneumococcal epidemiology has changed substantially as a result of conjugate vaccination. While PCVs have been highly effective in reducing the incidence of pneumococcal disease [4, 5, 11], continued vigilance is necessary to monitor for, and respond to, the emergence of potentially dangerous lineages not protected against by current vaccine formulations.

## Data bibliography

1. Sequence data from the 2001, 2004 and 2007 sampling periods were previously submitted to the European Nucleotide Archive (ENA) under project ERP000809.
2. Sequencing data from 2009 to 2011 have been deposited in NCBI SRA under BioProject PRJNA437866.
3. The pneumococcal capsular sequences were obtained from the database hosted by the Sanger Institute (https://www.sanger.ac.uk/resources/downloads/bacteria/streptococcus-pneumoniae.html), with the addition of the serotype 6C sequence from GenBank accession number EF538714.
4. Serotype 3 reference sequence: NCBI accession NC_017592.1.
